# Healthcare workers’ experiences of workplace violence: a qualitative study in Lebanon

**DOI:** 10.1186/s13031-023-00540-x

**Published:** 2023-10-03

**Authors:** Linda Abou-Abbas, Rana Nasrallah, Sally Yaacoub, Jessica Yohana Ramirez Mendoza, Mahmoud Al Wais

**Affiliations:** 1 International Committee of the Red Cross (ICRC), Beirut, Lebanon; 2 American University of Beirut, Beirut, Lebanon; 3 Université Paris Cité and Université Sorbonne Paris Nord, Inserm, INRAE, Center for Research in Epidemiology and Statistics (CRESS), Paris, France; 4 International Committee of the Red Cross, Geneva, Switzerland

**Keywords:** Healthcare workers, HCWs, Workplace violence, Qualitative study, Lebanon

## Abstract

**Background:**

The COVID-19 pandemic has brought unprecedented challenges to healthcare workers (HCWs) around the world. The healthcare system in Lebanon was already under pressure due to economic instability and political unrest before the pandemic. This study aims to explore the impact of COVID-19 and the economic crisis on HCWs’ experiences of workplace violence in Lebanon.

**Methods:**

A qualitative research design with an inductive approach was employed to gather data on workplace violence through Focus Group Discussions (FGDs) from HCWs in Tripoli Governmental Hospital (TGH), a governmental hospital in North Lebanon. Participants were recruited through purposive sampling. The interviews were conducted in Arabic, recorded, transcribed, and translated into English. Thematic analysis was used to analyze the data.

**Results:**

A total of 27 employees at the hospital participated in the six FGDs, of which 15 females and 12 males. The analysis identified four main themes: (1) Types of violence, (2) Events witnessed, (3) Staff reactions to violence, and (4) Causes of violence. According to the interviews conducted, all the staff members, whether they had experienced or witnessed violent behavior, reported that such incidents occurred frequently, ranging from verbal abuse to physical assault, and sometimes even involving the use of weapons. The study findings suggest that several factors contribute to the prevalence of violence in TGH, including patients’ financial status, cultural beliefs, and lack of medical knowledge. The hospital’s location in an area with a culture of nepotism and favoritism further exacerbates the issue. The staff’s collective response to dealing with violence is either to submit to the aggressor’s demands or to remove themselves from the situation by running away. Participants reported an increase in workplace violence during the COVID-19 pandemic and the exacerbated economic crisis in Lebanon and the pandemic.

**Conclusion:**

Interventions at different levels, such as logistical, policy, and education interventions, can help prevent and address workplace violence. Community-level interventions, such as raising awareness and engaging with non-state armed groups, are also essential to promoting a culture of respect and zero tolerance for violence.

## Introduction

Acts of violence in the workplace have far-reaching consequences that can disrupt various aspects of society [1]. Healthcare workers (HCWs) are often at a higher risk of being subjected to workplace violence, with up to 38% of HCWs experiencing violence at some point during their careers [2]. The prevalence of workplace violence (WPV) against HCWs was found to be high in Asian and North American countries, psychiatric and emergency department settings, and among nurses and physicians [3].

The COVID-19 pandemic has aggravated violence against HCWs [4–6], increasing existing sources of violence and opening new areas of confrontation between healthcare providers, patients, and their families [5]. From February to December 2020, the International Committee of the Red Cross (ICRC) received 848 reports of violence against HCWs related to COVID-19 across 42 countries. These incidents occurred in various regions around the world, including Europe, Africa, the Americas, and Asia [7]. A review of incidents from a lower-middle-income country revealed that the reasons for the assaults are varied, including unexpected outcomes or death of a patient, unavailability of resources at the hospital due to overcrowding, miscommunication, and a lack of awareness in society [8].

A joint study by several international organizations has found that violence against doctors is widespread and has increased since the start of the COVID-19 pandemic [5]. The study received responses from over 120 organizations and found that 58% of respondents perceived an increase in violence, with all respondents reporting verbal aggression, 82% mentioning threats and physical aggression, and 27% reporting staff being threatened by weapons [5]. The study highlights the need for concrete action to end impunity for those who are violent and suggests practical solutions, such as improving relations between health personnel and patients and implementing successful strategies from countries such as Bulgaria, Colombia, Italy, Portugal, and Taiwan. The study highlights the need to better understand how violence is affecting healthcare workforce and quality of services and take action to stop it [5].

WPV is a serious issue in Lebanon, and the healthcare sector is not immune to this problem. A study conducted in 2015 found that 62% of nurses in Lebanon experienced verbal abuse in the past year, while 10% reported physical abuse, including weapon attacks [9]. The economic crisis in Lebanon, combined with the ongoing COVID-19 pandemic for the past three years, has resulted in an increase in violent acts against HCWs, with hospitals becoming a target for frustrated individuals [10]. The situation is particularly challenging for Tripoli Governmental Hospital (TGH), which is the second largest public hospital in Lebanon where citizens suffer from low incomes and poverty. Its location is critical, as many armed clashes/hostilities take place in the surrounding area of the hospital, making it more vulnerable to workplace violence [11]. As HCWs play a critical role in providing essential services to the community and deserve to work in a safe and supportive environment, it is crucial to address the issue of workplace violence and gain a deeper understanding of the issues surrounding violence against healthcare providers in TGH. This study aims to understand HCWs’ perspectives on workplace violence, explore their preferences for interventions to prevent violence, and propose feasible methods to protect HCWs from violence. This research could be a crucial step towards improving the safety and well-being of HCWs in Lebanon and other similar settings.

## Methods

### Study design and setting

A qualitative research design with an inductive approach was employed to gather data on WPV through Focus Group Discussions (FGDs). The decision to initiate the research with FGDs rather than individual interviews was due to several factors including resource availability, research objectives, and the nature of the research question. Starting directly with FGDs was deemed efficient in terms of time and resources, especially when seeking a broader understanding of WPV by facilitating group interactions that stimulate participants to build on each other’s ideas and experiences. Additionally, FGDs can create an environment where participants feel more comfortable sharing sensitive or personal experiences due to the shared context and the support of the group.

The study was conducted at TGH that serves about 638,000 Lebanese (including 244,000 residents of Tripoli), 233,000 Syrian refugees, and roughly 50,000 Palestinian refugees. Approximately 400 healthcare providers (doctors and nurses) work at TGH [11].

### Participant recruitment

To ensure a diverse range of participants based on gender and occupations, we implemented a purposeful sampling procedure. This procedure involved contacting various categories of hospital staff and inviting them to participate in our study. Eligibility was extended to all staff members working within the hospital setting. Invitations to participate were conveyed through phone messages. Staff members who expressed their willingness to participate were subsequently contacted via phone messages to arrange the interview. Additionally, we meticulously planned the FGDs by predetermining the date, time, and location.

Our selection criteria focused mainly on individuals in direct contact with patients due to their unique vantage points and daily exposure to WP incidents providing firsthand perspectives on frontline dynamics. Additionally, administrative and support staff were included to contribute valuable insights into organizational aspects related to workplace violence, enriching our understanding of the broader context within healthcare organizations. These categories were considered most appropriate for our research, as they align closely with our research objectives, allowing us to gain comprehensive insights into WP in the healthcare setting.

The hospital staff who agreed to participate in the study were grouped according to their preferred time during the day.

Four FGDs were conducted for the study, as follows:

- A group of female nurses.
- Two groups of both female and male nurses.
- A group of hospital administrative staff.
- Two groups of other support staff including orderlies, lab technicians, cooks, housekeeping.

### Data collection

In February 2022, the FGDs were conducted by two investigators in Arabic in a private room at the hospital using a semi-structured interview guide (Appendix 1). Only non-identifiable information was collected and included gender and the participants’ job title (i.e., physician, nurse, paramedic). The interview guide included open-ended questions related to WPV, such as how it is defined, the forms it takes, examples of violent incidents, and the motives of perpetrators. Other questions included the staff’s reaction to the incidents and whether they could have reacted differently or prevented the event from happening. Training of HCWs, preventing violence, and hospital safety regulations were also discussed. The interviews lasted 45 min to an hour on average.

As we progressed through the study, we observed that new information and perspectives related to workplace violence became increasingly scarce. Instead, we encountered recurring themes and insights from participants, indicating that we had comprehensively explored the topic. This consistent repetition of information across participants signaled to us that we had achieved data saturation, where further data collection would likely yield diminishing returns in terms of new insights.

### Data gathering tool

The discussions were audio recorded as a means of capturing participants’ voices, experiences, and perspectives in their own words during the FGDs. Following the transcription, the original recordings were securely destroyed to uphold participants’ privacy and ensure the confidentiality of the information shared. This approach aligned with best practices in qualitative research to protect participants’ identities and uphold the integrity of the research process.

### Quality control and assurance

The research team rigorously ensured objectivity and impartiality in the formulation of research questions. Questions posed during interviews were deliberately crafted to be objective, avoiding any form of intervention or bias. The primary goal was to explore diverse dimensions of workplace violence and gather information essential for the study. Crucially, the interviewers maintained a neutral stance, refraining from expressing personal opinions or influencing participant responses. Importantly, no pre-existing relationships existed between the interviewers and participants, reinforcing the integrity of the research process. Data collection was conducted in a room within the hospital premises, selected for its convenience. This choice accommodated the participation of hospital staff during their work shifts, facilitating their engagement in the study. All staff members within the hospital, irrespective of their roles, were eligible to participate due to their direct interactions with patients, which made their perspectives valuable to the research objectives. The selection of participants was unbiased, guided solely by their roles in patient care and their exposure to workplace violence incidents.

### Ethical considerations

The approval of the Institutional Review Board (IRB) at American University of Beirut (AUB) (SBS-2021-0352) and the internal ethical review board at ICRC was obtained before starting the study (2109-APR). The study was conducted in accordance with ethical principles and guidelines, including informed consent, confidentiality, and the right to withdraw from the study at any time. The participants signed an informed consent form before the discussion, which emphasized the confidentiality of the information they shared. They were also informed that they could withdraw from participating in the study at any time.

### Data analysis

Audio-recorded FGDs were transcribed verbatim in the Arabic language. A rigorous manual analysis was undertaken to discern recurring themes, patterns, and insights pertaining to WPV experiences among HCWs. The verbatim transcripts were meticulously reviewed to extract pertinent concepts and phrases, which were then assigned as codes. These codes were subsequently organized into categories within a matrix structure. These categories aligned with overarching themes that were deduced from the research objectives and questions, allowing for a comprehensive exploration of WPV dimensions. The themes and sub-themes identified underwent thorough discussion within the research team to ensure accuracy and robustness. Quotes used in reporting findings were translated to English language.

## Results

A total of 27 employees at the hospital participated in the six FGDs, of which 15 females and 12 males. The participants were further categorized into three groups based on their occupations: nurses (14 participants), administrative staff (5 participants), and support staff (8 participants).

The analysis of the information gathered was conducted through a process of coding, sub-theme, and theme development. The coding scheme can be found in Table 1.

**Table 1:** Coding scheme

Main Themes	Sub-Themes	Codes
**Types of violence**	Verbal abuse	Shouting, Swearing, Derogatory Remarks, Cursing
	Physical Violence	Hitting, Kicking, Pushing
	Nonverbal or Subtle Forms of Violence	Body Language, Tone of Voice
	Weapons use and Threats	Use of Weapons, Threats
**Events Witnessed**	Verbal Abuse	Threats, Shouting, Cursing
	Physical Aggression	Punching, slapping, physical injury (bone fractures)
	Introduction of Weapons	Weapons brought into the hospital environment
**Staff reaction to violence**	Strategies for Dealing with Violence	Submission to aggressor’s demands, physically remove themselves from the situation, Running away to a safer location
	Fear and Avoidance	Fear of being attacked at work, Fear of being followed and harassed outside of work, Prioritizing self-protection
	Seeking External Assistance	Calling security guards, Asking for help from police
**Causes of violence**	Hospital related causes	Lack of security staff, Lack of intervention by army checkpoint, Laborious billing procedure for outpatients, Lack of clear visitation policy, Staff shortages, Inadequate facilities and resources
	Patient related causes	Financial stress of patients, Expectation of free treatment, long waiting times, Culture of favoritism, Insufficient medical knowledge of patients and families, Unrealistic expectations of healthcare staff, Misunderstanding of medical information

In the following paragraphs, each theme is described in more detail providing sample quotes, where appropriate.

### Types of violence

All participants unanimously agreed that any form of aggression experienced while performing their jobs in healthcare settings constitutes violence. This indicates a clear consensus among the participants regarding the definition of violence in the healthcare setting.

Based on the participants’ descriptions, the types of violence experienced in healthcare settings can be categorized into two main forms: verbal and physical. Verbal violence included any communication that is intended to harm or intimidate, such as shouting, swearing, or making derogatory remarks. Physical violence, on the other hand, included any intentional physical act that causes harm or injury, such as hitting, kicking, or pushing. Some participants also mentioned the potential for nonverbal or subtle forms of violence, such as body language or tone of voice, which can convey aggression or hostility. Additionally, some participants identified the use of weapons or threats as a form of violence. While most of the participants focused on the violence that they can face from the patients and their families, some mentioned that violence can be addressed from their colleagues as well. Moreover, it was acknowledged that violence in healthcare settings can also originate from staff members towards their patients.

### Events witnessed

All staff members have witnessed violence at work that ranged from verbal abuse such as being threatened, shouted at, and being cursed, to being punched or slapped and sometimes even physical injury in the form of bone fractures. It is important to note that the type of violence targeting males and females differed. Males were more likely to experience physical violence. In contrast, females were often targeted with verbal abuse, though they were not immune to physical violence either. Additionally, weapons were brought into the hospital and used against the staff, further exacerbating the risk and harm faced by everyone involved.

A nurse that was working in the Emergency Department (ED) was present during an event when a family member of a patient who was seeking care for a stabbing injury in the back was threatening to blow the ED with a bomb if his relative would have died or “*does not leave the hospital walking on his legs*”. He even shot the roof of the ED with the weapon he was holding.

The participant verbalized the following words:

“*It was one of the scariest moments of my life… my colleague and I had to help the bleeding patient, but we were hiding afraid to die… If my parents knew what I went through that day, they would have not allowed me to go to work again*”.

Another participant described being punched in the face by someone who came to the blood bank asking for O negative blood units. The lab technician ended up giving him a unit of blood from any type due to his fear. He mentioned:

*In times like that, all you think about is how to save yourself, your life, so that you remain available next to your family…*.

Almost half of the participants recalled a recent event experienced by a nurse at the Obstetrics and Gynecology (OBGYN) unit. The family members of a patient broke the fingers of the nurse for not being able to insert an IV line directly to the patient.

Administrative staff have also been subject to violence with four out of five having experienced violent episodes. The violence they encounter is primarily in the form of shouting and damage to the health facility and equipment causing destruction of glass and equipment in their vicinity.

“*We’re used to this kind of violence, we face it daily*”, they said.

Violence has been observed by staff across all categories, including those who do not have direct contact with patients and their families. For example, a cook working in the hospital’s kitchen was shouted at by a patient’s family member for not providing food, even though the patient was under medical orders not to eat due to a recent surgical operation. Additionally, a pharmacist was threatened with physical harm in the pharmacy department if they did not provide narcotics to an aggressive individual.

Some participants mentioned that verbal aggression between staff members may occur, but they are usually resolved immediately without further escalation. Additionally, one participant noted that in some cases, staff members may raise their voices and behave inappropriately towards patients and their families, which could be attributed to the high levels of stress they are experiencing.

“*We are all stressed, sometimes we shout at patients’ families or our colleagues due to the stress we are enduring inside and outside the hospital environment*”.

### Causes of violence

Staff reported the causes that could potentially lead to violent incidents in hospitals which can generally be divided into two categories: hospital-related and patient-related.

### Hospital-related

One of the main causes that staff members at the hospital cited for potential violence was the inadequate number of security guards. With only two guards stationed at the entrance of the hospital, there was concern that they would not be able to effectively respond to any violent incidents that might occur. Additionally, even though at the hospital’s parking premises there is an army checkpoint; they are not authorized to intervene in such situations, further exacerbating the security issue.

Another reason mentioned by the administrative staff was the laborious and protracted billing procedure for outpatients. To bill the patients, the paperwork needs to be physically transported across several departments, such as pharmacy, laboratory, and imaging, which is a manual process. This process is time-consuming which adds frustration to the patient and his family and can sometimes escalate into violence.

The lack of a clear visitation policy was also a concern raised by nurses at the hospital. Without clear restrictions on who can visit patients and when, anyone can enter the hospital at any time, including individuals who may be carrying weapons.

### Patient-related

According to the TGH staff, the main reason of violence in the hospital is attributed to the financial status of the patients. As a public hospital, many patients expect to receive free treatment. However, when informed of the costs associated with their care by the admitting department, they become overwhelmed and agitated, which can escalate to violent behavior. Additionally, the hospital’s location in an area with a culture of favoritism contributes to some patients’ belief that they can obtain special treatment by shouting and threatening, which may also contribute to incidents of violence in the hospital.

One of the staff said that “*the clients of the hospital know that if they shout and threaten, they will get whatever they want*”.

The insufficient medical knowledge of patients and their families is identified by almost all participants as a significant factor contributing to violence in TGH. Due to their limited understanding of the disease, patients and their families have unrealistic expectations of the healthcare staff’s ability to maintain the patient’s life, which can escalate to violent conduct. Furthermore, the COVID-19 pandemic has worsened this situation, as participants noted that the lack of comprehension of this novel disease has also played a role in violent incidents.

“*Families and patients do not understand why they cannot see their relative at isolation, and that makes them aggressive*”.

### Staff reactions to violence

The staff collectively agreed that the best way to deal with violence is to either submit to the aggressor’s demands to avoid being subjected to violence or to physically remove themselves from the situation by running away. Two nurses working at the pharmacy department described how nurses from the Obstetrics and Gynecology department ran away from their unit to the pharmacy department when they were aggressed by a patient’s family.

Staff members in hospitals often avoid reacting or intervening in violent situations due to their fear of not only being attacked at work but also being followed and harassed on their way to and from work, as they mentioned:

“*In these situations, we just need to protect ourselves… we agree with whatever the aggressor says and do whatever he asks for*”.

The response to violence differs between males and females. Males tend to face the perpetrator and confront them directly, possibly reflecting societal expectations of male protectiveness or assertiveness. In contrast, females tend to prioritize escape and avoidance, preferring not to engage with the perpetrators directly. They may even respond to the perpetrators’ needs, even if those needs are not relevant or urgent, as a means of defusing the situation. Some female staff members mentioned that when a perpetrator attacks the nursing station or arrives angry at a department, their aggression often subsides upon realizing that the entire staff present is female. This observation suggests that the gender composition of the staff can have an impact on the dynamics of the situation, potentially leading to a de-escalation of the aggression.

In cases of violence, staff members seek assistance by calling the few available security guards at the hospital or asking for help from the police, recognizing the importance of external support in managing violent incidents and ensuring the safety of all involved parties.

## Discussion

The study conducted sheds light on the alarming issue of violence against HCWs in TGH. According to the interviews conducted, all the staff members, whether they had experienced or witnessed violent behavior, reported that such incidents occurred frequently, ranging from verbal abuse to physical assault, and sometimes even involving the use of weapons. The study findings suggest that several factors contribute to the prevalence of violence in TGH, including patients’ financial status, cultural beliefs, and lack of medical knowledge. The hospital’s location in an area with a culture of clout and favoritism further exacerbates the issue. The staff’s collective response to dealing with violence is either to submit to the aggressor’s demands or to remove themselves from the situation by running away. In this discussion section, we will examine the implications of these findings and propose recommendations to address this problem.

Our findings are consistent with a recent meta-analysis of 38 studies involving 63,672 healthcare workers (HCWs), which reported high prevalence rates of workplace violence (WPV) among HCWs. The analysis revealed significant rates of physical violence (9%), verbal violence (48%), and emotional violence (26%) among HCWs. Furthermore, the meta-analysis indicated an escalation of WPV, physical violence, and verbal violence during the mid- to late-stages of the COVID-19 pandemic [12]. These findings emphasize the critical need to address WPV and prioritize the well-being and safety of HCWs. The patients’ financial status appears to be a significant contributor to violent behavior, as many patients expect to receive free treatment at TGH, being a public hospital. However, they become agitated when informed of the costs associated with their care, which can escalate to violent conduct. The cultural beliefs and attitudes of patients towards the hospital staff also play a role in the occurrence of violence. Patients who believe that shouting and threatening will give them preferential treatment may become violent when their expectations are not met. The lack of medical knowledge among patients and their families is also a significant factor contributing to violent behavior. Patients and their families may have unrealistic expectations of the healthcare staff’s ability to maintain the patient’s life due to their limited understanding of the disease. The COVID-19 pandemic has further exacerbated the issue of violence in the hospital, with participants reporting that the lack of knowledge about the new disease has contributed to violent incidents. Working with people infected with COVID-19 is also a factor for violence [6]. The weakness of the security logistics at the hospital has also been a major reason for violence. The issues of corruption in Lebanon have also affected violence in the TGH. Many participants mentioned that people who commit violence against HCWs at the hospitals are usually covered by political parties. They threat with weapons and use them in the hospital knowing that eventually, there will be no punishment for their actions. The fact that TGH is a public hospital makes it a “punching bag” for the Lebanese patients that are frustrated from the Lebanese Government, so they pour their anger against the corrupted system in Lebanon on the healthcare workers at the hospital.

Differences were observed between males and females in terms of the types of violent incidents witnessed and the corresponding reactions exhibited. Males are more likely to witness and experience physical violence, such as being punched, slapped, or sustaining physical injuries. This could be attributed to societal expectations of male dominance and the perceived need for physical confrontation. On the other hand, females are more likely to encounter verbal abuse and emotional violence. When faced with violence, males tend to confront the perpetrators directly, possibly driven by societal norms of masculinity and the desire to protect themselves or others. In contrast, females often prioritize their safety by opting for escape and avoiding direct confrontation. They may comply with the aggressor’s demands to de-escalate the situation or minimize the risk of harm. These gender-specific responses may be influenced by social conditioning and self-preservation instincts, highlighting the complex interplay between societal expectations, gender roles, and individual coping mechanisms in the face of violence. However, it is important to note that these findings should not overshadow the fact that violence can affect individuals of all genders and that the experiences of individuals may vary widely. Each case should be considered on its own merits, and it is crucial to avoid making broad generalizations based solely on gender. Addressing violence requires comprehensive efforts that focus on prevention, support for survivors, and challenging harmful societal norms and behaviors.

It’s important to note that not all HCWs initially approached for participation in our study agreed to participate to the study. Possible reasons are unavailability during the study period or may be concerns related to the sensitivity of the topic, given that workplace violence is a complex and sensitive issue. We recognize that their non-participation introduces certain limitations and potential biases as their perspectives and experiences, which could have enriched our findings, are not represented. Consequently, we have taken great care to accurately present the data collected from willing participants in a manner that faithfully reflects their experiences within the study’s scope.

Interventions should be implemented promptly to enhance the security measures in hospitals, given the severity of the issue of violence against staff members. To improve security measures at hospitals, various interventions can be implemented at the organizational level. Logistical interventions, policy initiation interventions, and staff education can help prevent workplace violence. One effective logistical intervention is to install metal doors with access restricted to staff ID cards at hospital entrances and unit doors. Additionally, increasing the number of security guards and placing at least one guard on each hospital floor can help limit the number of visitors and prevent unwanted access. Metal detectors at the main entrance can also help prevent visitors from entering the hospital with weapons. At the policy level, visitation restrictions can be implemented, such as limiting visits to two family members per patient. Staff education and training programs can be conducted to prevent and manage workplace violence. Research has shown that staff training for violence prevention and management can reduce the consequences of violence [13]. Healthcare organizations, policymakers, and the government should work together to implement these interventions to ensure that healthcare workers can provide care safely and without fear of violence. Staff have shown willingness to participate in such training during focus group discussions.

At the community level, raising awareness among the adjacent population about the importance of respecting the hospital’s facilities and staff is one such intervention. This can help the community understand the crucial role of healthcare workers in treating and preventing diseases and promote their protection instead of violation. Another important intervention is to engage with non-State armed groups in the area to prevent violence against healthcare workers. The International Committee of the Red Cross (ICRC) has set an example in 2014 by counseling and meeting with them and signing an agreement to avoid interfering in the hospital’s work and protecting healthcare workers [7]. These interventions involve all stakeholders in the problem and have shown positive impacts in reducing violence against healthcare workers in recent studies [13].

## Conclusion

Violence against healthcare workers is a critical issue that affects the quality of healthcare services and the safety of both HCWs and patients. Our findings, derived from the perspectives of healthcare workers (HCWs), suggest that the problem of violence against HCWs is multifaceted, with various factors contributing to its occurrence. These factors include patient-related, organizational, and community-related factors. Interventions at different levels, such as logistical, policy, and education interventions, can help prevent and address workplace violence. Community-level interventions, such as raising awareness and engaging with non-state armed groups, are also essential to promoting a culture of respect and zero tolerance for violence. It is crucial for all stakeholders, including healthcare organizations, policymakers, the government, and the community, to work together to implement these interventions to ensure that healthcare workers can provide care safely and without fear of violence or harm.

## Disclaimer

The authors confirm that the views and opinions expressed in this publication do not in any way constitute the official view or position of the ICRC. Every effort has been made to comply with our duties of discretion regarding activities undertaken during our employment/missions with the ICRC.

## Data Availability

The data collected for this qualitative study is not publicly available due to the confidential nature of the information shared by participants. Access to the data is restricted to the research team to maintain privacy and ensure compliance with ethical guidelines.

## Appendix 1

**Interview Topic guide**.

A. **Introduce yourself, provide the consent form**.
B. **Collect Demographic information: Gender & job title**.
C. **Workplace Violence**.

1. How do you define occupational violence (i.e., workplace violence)? In what forms does it occur? Can you give examples from your experience (whether you witnessed violence or got exposed to it)?
2. Have you ever been exposed to violence at work/healthcare setting?
3. Why do you think such aggressive incidents take place? What are the motives of the perpetrator?
4. How did you react to the incidents that you got exposed to or witnessed? And do you think you could have reacted differently or maybe prevented the event from happening?
5. Do you think training of healthcare workers in communication/counseling skills, training in managing violence … would help prevent violent incidents?
6. Do you think it would be useful to increase resources in combating violence; specifically, by increasing security personal and facilities, working conditions and incentives for healthcare workers, and adequate facilities (equipment/medicines/ healthcare workers)?
7. What rules and regulations are needed to ensure that the environment is safe at the hospital?
8. How willing are you to engage in specific programs to combat violence? Why are you encouraged and why not?

## Abbreviations

COVID-19: Coronavirus disease-2019
HCWs: Health care workers
FGD: Focus group discussion
TGH: Tripoli Governmental Hospital
ICRC: International Committee of the Red Cross
WPV: Workplace violence

## References

[1] Liu J (2019). Workplace violence against nurses, job satisfaction, burnout, and patient safety in chinese hospitals. Nurs Outlook.

[2] WHO, World Health Organization., Preventing violence against health workers, available: https://www.who.int/activities/preventing-violence-against-health-workers. 2022.

[3] Liu J (2019). Prevalence of workplace violence against healthcare workers: a systematic review and meta-analysis. Occup Environ Med.

[4] Elsaid NMAB (2022). Violence against healthcare workers during coronavirus (COVID-19) pandemic in Egypt: a cross-sectional study. Egypt J Forensic Sci.

[5] Thornton J (2022). Violence against health workers rises during COVID-19. The Lancet.

[6] Bitencourt MR (2021). Predictors of violence against health professionals during the COVID-19 pandemic in Brazil: a cross-sectional study. PLoS ONE.

[7] ICRC, International Committee of the Red Cross., COVID-19 Special, Available: https://healthcareindanger.org/covid-19-special/?__hstc=246532443.c138db4f20418b575032677d21f6d14a.1669035781842.1669040887977.1679927932677.3&__hssc=246532443.1.1679927932677&__hsfp=1617094012. 2020.

[8] Bhatti OA (2021). Violence against Healthcare Workers during the COVID-19 pandemic: a review of incidents from a Lower-Middle-Income Country. Annals of Global Health.

[9] Alameddine M, Mourad Y, Dimassi H (2015). A National Study on Nurses’ exposure to Occupational Violence in Lebanon: prevalence, Consequences and Associated factors. PLoS ONE.

[10] Fleifel M, Abi Farraj K (2022). The lebanese Healthcare Crisis: an infinite calamity. Cureus.

[11] AFD, Reinforcing the provision of care at Tripoli Governmental Hospital (TGH.), available: https://www.afd.fr/en/carte-des-projets/lebanon-reinforcing-provision-care-tripoli-governmental-hospital. 2022.

[12] Zhang S et al. Workplace violence against healthcare workers during the COVID-19 pandemic: a systematic review and meta-analysis. Environ Sci Pollut Res Int, 2023: p. 1–15. 10.1007/s11356-023-27317-2 PMC10199297 37209334

[13] Bellizzi S (2021). Violence against Healthcare: a Public Health Issue beyond Conflict Settings. Am J Trop Med Hyg.

